# Estimating the effect of hepatitis C infection on multidrug-resistant tuberculosis treatment outcomes under hypothetical interventions on regimen composition and adherence

**DOI:** 10.1093/aje/kwag024

**Published:** 2026-02-02

**Authors:** Allison LaHood, James Robins, Helen R Stagg, Sara Sauer, Saman Ahmed, Mathieu Bastard, Lorenzo Guglielmetti, Catherine Hewison, Helena Huerga, Afshan K Isani, Palwasha Khan, Uzma Khan, Nino Lomtadze, Shahid Mamsa, Nara Melikyan, Carole D Mitnick, Michael L Rich, Kwonjun Seung, Muhammad Rafi Siddiqui, Alena Skrahina, Assel Stambekova, Girum B Tefera, Molly F Franke

**Affiliations:** Department of Epidemiology, Harvard T.H. Chan School of Public Health, Boston, MA 02115, United States; Department of Global Health and Social Medicine, Harvard Medical School, Boston, MA 02115, United States; CAUSALab, Department of Epidemiology, Harvard T.H. Chan School of Public Health, Boston, MA 02115, USA; Department of Infectious Disease Epidemiology, London School of Hygiene & Tropical Medicine, London WC1E 7HT, United Kingdom; Department of Global Health and Social Medicine, Harvard Medical School, Boston, MA 02115, United States; Interactive Research and Development (IRD), Karachi 75190, Pakistan; Field Epidemiology Department, Epicentre, Paris 75019, France; Department of Infectious, Tropical Diseases and Microbiology, IRCCS Sacro Cuore Don Calabria Hospital, Negrar di Valpolicella, Verona, Italy; Médecins Sans Frontières, Paris 75019, France; Field Epidemiology Department, Epicentre, Paris 75019, France; Centers for Disease Control and Prevention, Directorate General Health Services, Sindh, Pakistan; Interactive Research and Development (IRD) Global, 048581, Singapore; Department of Clinical Research, Faculty of Infectious and Tropical Diseases, London School of Hygiene & Tropical Medicine, London WC1E 7HT, United Kingdom; Department of Epidemiology, Biostatistics, and Occupational Health, McGill University, Montreal, QC H3A 1A2, Canada; The National Center for Tuberculosis and Lung Diseases, Tbilisi 0101, Georgia; Indus Hospital & Health Network (IHHN), Karachi 75190, Pakistan; Field Epidemiology Department, Epicentre, Paris 75019, France; Department of Global Health and Social Medicine, Harvard Medical School, Boston, MA 02115, United States; Partners In Health, Boston, MA 02199, United States; Division of Global Health Equity, Brigham and Women's Hospital, Boston, MA 02115, United States; Department of Global Health and Social Medicine, Harvard Medical School, Boston, MA 02115, United States; Partners In Health, Boston, MA 02199, United States; Division of Global Health Equity, Brigham and Women's Hospital, Boston, MA 02115, United States; Department of Global Health and Social Medicine, Harvard Medical School, Boston, MA 02115, United States; Partners In Health, Boston, MA 02199, United States; Division of Global Health Equity, Brigham and Women's Hospital, Boston, MA 02115, United States; Institute of Chest Diseases (ICD), Kotri 76040, Sindh, Pakistan; Republican Scientific and Practical Center of Pulmonology and Tuberculosis, 220053 Minsk, Belarus; Partners In Health, Almaty, Kazakhstan; Partners In Health, Freetown, Sierra Leone; Department of Epidemiology, Harvard T.H. Chan School of Public Health, Boston, MA 02115, United States; Department of Global Health and Social Medicine, Harvard Medical School, Boston, MA 02115, United States

**Keywords:** multidrug-resistant tuberculosis, rifampicin-resistant tuberculosis, hepatitis C, treatment adherence, losses-to-follow-up

## Abstract

Hepatitis C virus (HCV) infection is associated with unfavorable multidrug- and rifampicin-resistant (MDR/RR) tuberculosis (TB) outcomes. We examined whether this association would decrease in settings where no participants were lost-to-follow-up or where all adhered to regimens comprised of priority TB drugs. We analyzed data from 1,530 participants with HCV testing in the endTB observational cohort (NCT03259269). We estimated the relative risk of death, treatment failure, and loss-to-follow-up comparing participants with and without HCV, using inverse probability weighting to adjust for confounding. We then estimated relative risks of HCV on death and failure in weighted pseudopopulations representing hypothetical interventions eliminating loss-to-follow-up and ensuring adherence to strong MDR/RR-TB regimens. The unadjusted risk difference comparing participants with and without HCV was 14.1% (95% confidence interval [CI], 8.0%-20.1%), decreasing to 11.0% (95% CI, 3.0%-19.1%) after weighting. In pseudopopulations without loss-to-follow-up or with adequate adherence to strong regimens, the risk differences were 7.7% (95% CI, 0.8%-16.2%) and 7.0% (95% CI,−1.6% to 17.3%), respectively. Adjustment for baseline confounders attenuated the association between HCV and unfavorable outcomes, suggesting these factors partly explain the disparity. Further attenuation after eliminating loss-to-follow-up suggests that improving treatment retention in MDR/RR-TB care may reduce outcome disparities among patients with HCV.

## Key points

- We aimed to understand why HCV infection is associated with poor drug-resistant tuberculosis treatment outcomes and whether hypothetical interventions on regimen composition and adherence might mitigate the disparity. We found that baseline characteristics partly explain the disparity in outcomes by HCV status, and preventing losses to follow-up may reduce this disparity. Among patients with drug-resistant tuberculosis, efforts to reduce disparities in treatment outcomes between patients with and without HCV should include safeguards to prevent losses-to-follow-up.

## Introduction

Hepatitis C virus (HCV) infection is associated with unfavorable multidrug- and rifampicin-resistant (MDR/RR) tuberculosis (TB) outcomes, but the reasons why are not well described. One possibility is that baseline differences between people with and without HCV infection (eg, age, sex, marital status, employment, substance use, malnutrition, and severity of TB disease^1^^-^^4^), rather than HCV infection itself, explain this association. Alternatively, HCV could directly impact TB treatment outcomes by increasing the risk of anti-TB drug–induced hepatotoxicity^5^^,^^6^ prompting treatment interruptions or altering drug metabolism. Additional explanations include differential TB medication prescribing practices by health providers and differential adherence to TB medications by individuals undergoing treatment. The extent to which prescribing practices and medication adherence contribute to the relationship between HCV and treatment outcomes is particularly relevant because these factors are intervenable in clinical practice.

While international recommendations, including those endorsed by the World Health Organization (WHO), identify priority drugs for treating MDR/RR-TB, the specific combination and number of drugs prescribed varies depending on clinical discretion, including consideration of a patient’s medical history. For example, one 2015 study in New York City found that patients with TB and concurrent HCV disease were more likely to receive weaker, liver-sparing anti-TB regimens compared to those without HCV.^7^ Although new and repurposed drugs currently prioritized for MDR/RR-TB treatment are less hepatotoxic than past recommended regimens, most drugs, including highly effective prioritized drugs, such as bedaquiline and fluoroquinolones (levofloxacin and moxifloxacin), cause hepatotoxicity.^8^ If individuals with HCV are less likely to receive a potent regimen, interventions focused on constructing safe and effective regimens for this group might mitigate unfavorable MDR/RR-TB end-of-treatment outcomes.

Differential adherence to anti-TB medication and loss-to-follow-up are additional treatment factors that potentially drive unfavorable outcomes among patients with HCV. Socioeconomic factors associated with HCV infection, such as substance use^9^^-^^11^ and homelessness,^12^ are risk factors for suboptimal adherence. Suboptimal adherence, in turn, increases the risk of treatment failure and TB recurrence. Interventions to improve adherence, such as psychosocial support, community-based care, and adherence support, are effective at improving outcomes and mitigating loss-to-follow-up.^13^^-^^15^ Despite a strong theoretical basis for suboptimal adherence as a mediator of the relationship between HCV and treatment outcomes, the extent to which adherence contributes is unclear.

In this study, we employed methods rooted in causal inference to elucidate the relationship between HCV and MDR/RR-TB end-of-treatment outcomes. Specifically, we aimed to estimate the effect of HCV on outcomes, adjusting for key confounders. To evaluate the impact of hypothetical interventions on (1) loss-to-follow-up and (2) inadequate adherence to strong MDR/RR-TB regimens, we examined whether any of the effect of HCV on death and failure would be mitigated in settings where (1) no one was lost-to-follow-up or (2) all individuals received and adhered to a treatment regimen comprised of priority TB drugs.

## Methods

### Study population

We used data from the endTB observational cohort (clinicaltrials.gov NCT03259269). From 2015 to 2018, endTB prospectively enrolled individuals from 17 countries who were prescribed 18- to 24-month individualized regimens that included bedaquiline and/or delamanid and were constructed according to local programmatic standards informed by endTB clinical guidance and WHO guidelines.^16^^-^^18^ Standardized clinical forms were used for data collection throughout participation. Medical providers referenced the endTB clinical guide^18^ as a resource for clinical decision making, and adverse events were monitored by a central pharmacovigilance team. Comprehensive details can be found in the published study protocol.^19^

### Definition of HCV exposure and MDR/RR-TB outcomes of interest

Participants with a positive baseline antibody test, polymerase chain reaction (PCR), or viral load were considered positive for HCV infection. The vast majority (96%) of included participants received an antibody test because viral load and PCR testing was inconsistently available across study sites. The WHO defines unfavorable TB end-of-treatment outcomes as death, treatment failure, or loss-to-follow-up. Also of interest for this analysis was a composite outcome of death and treatment failure, which may be interpreted as TB-specific outcomes.^20^

### Calculating and classifying adherence

During treatment, adherence was reported on treatment cards by a health facility supervisor during daily direct observed therapy (DOT) or self-reported by the participant. Adherence rate was calculated as the number of days with complete adherence to all drugs in the regimen divided by the number of prescribed days in that month. We classified individuals as having adequate adherence in a given month if they had an adherence rate greater than or equal to 80%.

### Definition of a strong regimen

We defined a regimen as strong if it contained all three group A drugs (bedaquiline, linezolid, and one fluoroquinolone between levofloxacin or moxifloxacin), or two group A drugs and at least one likely effective group B drug (clofazimine and cycloserine or terizidone).^21^ This definition was based on an individual patient data meta-analysis of several randomized controlled trials and observational studies.^22^^,^^23^ The individual composition of a strong regimen could vary over time, so long as it met the definition. The one exception was bedaquiline, which had a minimum duration of 6 months (ie, after 6 months, it counted toward the strong regimen definition for the entire duration of treatment).^21^^,^^24^^,^^25^ Drugs counted toward a strong regimen only if they were likely to be effective in the participant based on (1) documented susceptibility from drug susceptibility testing or (2) in the absence of resistance testing, no prior treatment with the drug for one month. Any likely effective drugs prescribed on the 14th day after enrollment were included in the baseline regimen composition. We selected day 14 to allow for early adjustments to treatment regimens.

### Eligibility criteria

We included participants who enrolled in the endTB observational study within one month of initiating programmatic treatment for MDR/RR-TB and with a baseline HCV antibody test, PCR, or viral load result. We excluded participants from countries with inconsistent adherence data and countries with no recorded positive HCV test results. The small number of participants who transferred out of the study or whose outcomes were not evaluated were also excluded.

### Missing data

We addressed missing data in categorical baseline and time-varying covariates by adding missing indicators. We imputed missing monthly adherence data by averaging the adherence rates from months flanking the missingness. If there were no consecutive flanking months, we imputed the mean monthly adherence for that participant to the missing month(s). Most study countries had < 5% overall missingness in longitudinal adherence data, with the exception of three study countries (Ethiopia, Indonesia, and Pakistan) with substantial (>25%) missingness in monthly adherence data. We defined a binary variable for whether a participant was from Ethiopia, Indonesia, or Pakistan versus other countries to adjust for between-country differences in adherence reporting.

### Statistical analysis

Using causal structure informed by subject matter knowledge (Figure S1) to guide confounding adjustment, we estimated the total effect of HCV on death, failure, and loss-to-follow-up, using inverse-probability (IP) weighting to adjust for baseline confounding. We then estimated the controlled direct effects (described below) in pseudopopulations where (1) no one was lost-to-follow-up and (2) all individuals adhered to strong regimens throughout treatment. All analyses were conducted in R version 4.3.1 (R Core Team, 2023).

### Estimation of the total effect of HCV on unfavorable outcomes

We first estimated the crude association of HCV on death, treatment failure, and loss-to-follow-up by fitting a logistic regression to obtain unadjusted estimates of the absolute risks, risk difference, and risk ratio for participants with and without HCV. Then, we constructed IP of HCV weights by fitting a logistic regression predicting HCV infection conditional on baseline confounders (sex, age, HIV, low BMI, intravenous drug use, alcohol use, unemployment, partnership status, cavitary disease, smear positive results, and culture positive results). We then fit a weighted logistic regression outcome model to obtain the marginal total effect (ie, the relative risk of unfavorable outcomes comparing participants with and without HCV). We estimated standardized mean predicted probabilities of unfavorable outcomes if everyone had HCV and if no one had HCV and compared them via risk differences and risk ratios.

### Estimation of the controlled direct effects of HCV on death and failure

We fit two IP weighted models to estimate the controlled direct effects of HCV on death and failure had (1) no one been lost-to-follow-up and (2) had everyone adhered to a strong MDR/RR-TB regimen. To estimate the former, we censored participants when they were lost-to-follow-up and used the product of IP of censoring weights and the IP of HCV weights to define a pseudopopulation where no one was lost to follow-up.

We then estimated the controlled direct effect of HCV infection on death and failure in an IP-weighted pseudopopulation where all participants were prescribed and adhered to a strong regimen (ie, monthly adherence was always at least 80%, and nobody was lost-to-follow-up). We censored participants who were not prescribed a strong baseline regimen and who experienced any of the following events, at their first occurrence: (1) suboptimal adherence for two consecutive months, (2) discontinuation of a strong regimen for non-clinical reasons (eg, administrative issues, medication stockouts) for two consecutive weeks, and (3) outcome of lost-to-follow-up. We chose a two-week period for regimen discontinuation because it aligns with prior analyses.^25^^-^^27^

To estimate time-varying censoring weights for both analyses, we fit pooled logistic regression models to predict whether participants remained uncensored, conditional on HCV, aforementioned baseline confounders and time-varying predictors of censorship and death and failure (sputum smear and culture results and cavitary disease). Full details on weight derivation are provided in supplemental Table S5. We then fit weighted logistic regression models and estimated absolute risks among participants with and without HCV and the risk differences and risk ratios by standardizing mean predicted probabilities of death and failure if everyone had HCV and if no one had HCV. Non-parametric bootstrapping with 1,000 resamples was used to calculate 95% CIs for all estimates.

## Results

**Figure 1:**
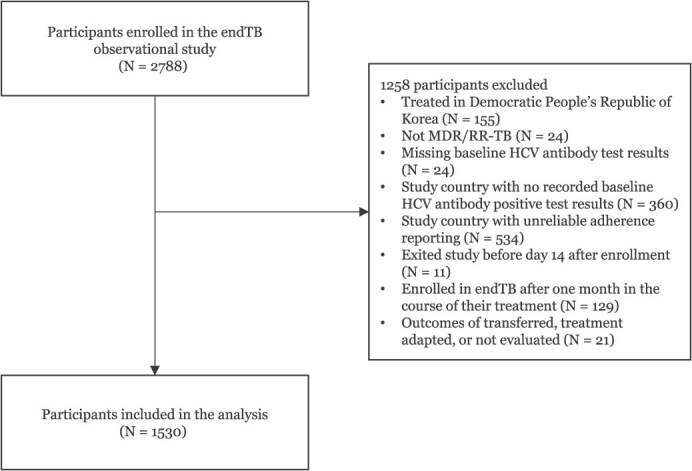
Flowchart of participants included in the analysis in the endTB observational study cohort.

During the enrollment period, 2,788 participants consented to participate in the endTB observational study and initiated treatment (Figure 1). We excluded 155 participants from the Democratic People's Republic of Korea due to differences in study procedures and regimens. Twenty-four participants who did not have MDR/RR-TB, 24 with missing baseline HCV test results, and 360 from study countries with no recorded baseline HCV-positive test results were excluded. Additionally, we excluded 534 participants from nine study countries with inconsistent adherence reporting, 11 participants who exited the study before day 14, and 129 who enrolled in the study after 1 month of treatment. Twenty-one participants with outcomes of transferred or not evaluated were excluded. Overall, 1,530 participants were eligible for our analysis.

### Baseline characteristics and end-of-treatment outcomes

The majority of the analysis population underwent treatment in Kazakhstan (43%), Pakistan (17%), and Georgia (16%) (Table S1). Participants with baseline HCV infection were generally older, more likely to be male (84% versus 60% without HCV) and use substances, had a higher prevalence of HIV (17% compared to 3%), indicators of social vulnerability (homelessness, unemployment, refugee, and incarceration), and uncoupled civil status (unmarried or living with a partner) (Table 1). Participants with HCV infection more frequently experienced outcomes of death, treatment failure, and lost-to-follow-up (21% died or experienced failure and 13% were lost to follow-up) compared to participants without HCV (12% died or experienced failure and 7.5% were lost to follow-up) (Table S2). The majority (77%) of treatment failures were due to microbiologic failure (Table S3). The univariate risk ratio for the association between HCV infection and hepatotoxicity was 2.24 (95% CI, 1.43-3.20) (Table S4).

**Table 1:** Baseline characteristics of eligible participants (*n* = 1,530).

**Characteristics**	***N*** ^**a**^	**HCV test results**		**Overall, *n* = 1530**
		**Positive *n* = 256**	**Negative *n* = 1274**	
		***n* (%)**	***n* (%)**	***n* (%)**
Female	1530	40 (15.6%)	504 (39.6%)	544 (35.6%)
Age in years, mean (SD)	1530	42.0 (9.7)	36.3 (13.3)	37.3 (12.9)
Malnutrition (BMI < 18.5)	1521	75 (29.5%)	542 (42.8%)	617 (40.6%)
HIV	1530	43 (16.8%)	40 (3.1%)	83 (5.4%)
Diabetes	1528	36 (14.2%)	188 (14.8%)	224 (14.7%)
Liver injury	1453	8 (3.3%)	5 (0.4%)	13 (0.9%)
Cavitary disease	1426	164 (68.3%)	779 (65.7%)	943 (66.1%)
Smear grade at treatment initiation	1430			
Negative		79 (32.6%)	516 (43.4%)	595 (41.6%)
Scanty 1-3		4 (1.7%)	8 (0.7%)	12 (0.8%)
Scanty 4-9		8 (3.3%)	38 (3.2%)	46 (3.2%)
One plus		64 (26.4%)	337 (28.4%)	401 (28.0%)
Two plus		40 (16.5%)	165 (13.9%)	205 (14.3%)
Three plus		47 (19.4%)	124 (10.4%)	171 (12.0%)
Positive culture at treatment initiation	1407	171 (70.7%)	746 (64.0%)	917 (65.2%)
Pulmonary disease	1528	256 (100.0%)	1260 (99.1%)	1516 (99.2%)
Resistance profile	1530			
Not tested for MDR/RR		1 (0.4%)	15 (1.2%)	16 (1.0%)
MDR/RR-TB with FQ resistance		58 (22.7%)	329 (25.8%)	387 (25.3%)
MDR/RR-TB with Inj resistance		31 (12.1%)	190 (14.9%)	221 (14.4%)
MDR/RR-TB with FQ and inj both resistant		122 (47.7%)	472 (37.0%)	594 (38.8%)
MDR/RR-TB without resistance to FQ/inj		35 (13.7%)	217 (17.0%)	252 (16.5%)
MDR/RR-TB without testing to FQ and inj		9 (3.5%)	51 (4.0%)	60 (3.9%)
Smoker	1488	155 (63.0%)	375 (30.2%)	530 (35.6%)
Uses alcohol	1493	69 (27.5%)	141 (11.4%)	210 (14.1%)
Uses intravenous drugs	1472	37 (15.7%)	6 (0.5%)	43 (2.9%)
Employed	1518	43 (16.9%)	463 (36.7%)	506 (33.3%)
Incarcerated	1262	103 (46.0%)	121 (11.7%)	224 (17.7%)
Homeless	1467	14 (5.8%)	54 (4.4%)	68 (4.6%)
Refugee	1494	14 (5.7%)	41 (3.3%)	55 (3.7%)
Married or living with partner	1521	129 (51.0%)	699 (55.1%)	828 (54.4%)
Prescribed strong MDR/RR-TB regimen	1530	164 (64.1%)	893 (70.1%)	1057 (69.1%)
Number of group A drugs, mean (SD)	1530	1.8 (0.7)	2.0 (0.7)	2.0 (0.7)
Number of group B drugs, mean (SD)	1530	0.8 (0.5)	0.9 (0.5)	0.9 (0.5)
Number of group C drugs, mean (SD)	1530	1.3 (0.9)	1.3 (1.0)	1.3 (0.9)

Abbreviations: DAAs, direct-acting antivirals; FQ, fluoroquinolone; HCV, hepatitis C virus; Inj, injectable; MDR/RR-TB, rifampin-resistant/multidrug-resistant tuberculosis; SD, standard deviation.

^a^Number of individuals with available data in each characteristic.

### Description of uncensored participants

**Figure 2:**
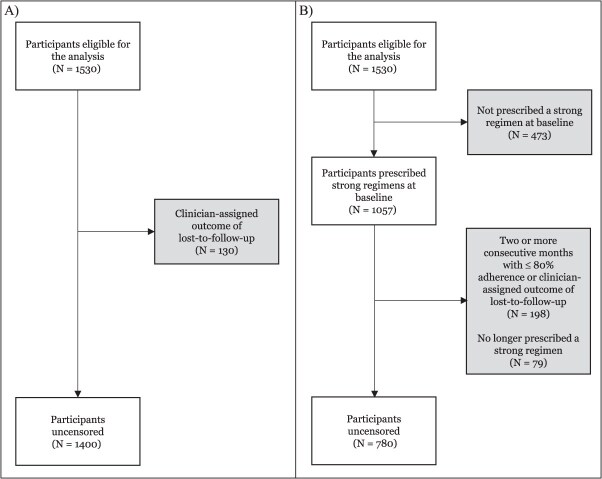
Flowchart of censoring in inverse probability weighted models (A) due to lost-to-follow-up only and (B) due to baseline regimen composition, suboptimal adherence, lost-to follow-up, and discontinuation of a strong regimen.

Of the 1,530 participants analyzed, 1,400 (91.5%) remained uncensored in the analysis, where we censored participants who were lost to follow-up (Figure 2A). In the analysis that censored participants who did not adhere to strong regimens, 473 (30.9%) were not prescribed a strong baseline regimen, 198 (15.0%) had suboptimal adherence or were lost to follow-up, and 79 (7.5%) discontinued a strong regimen for non-clinical reasons. Ultimately, 780 of the 1,530 participants (51.6%) adequately adhered to a strong regimen throughout treatment (Figure 2B).

### Total effect of HCV on unfavorable outcomes

The unadjusted risk of unfavorable outcomes (death, failure, and lost-to-follow-up) among those with HCV was 34.0% (95% CI, 28.3-39.5) compared to 19.9% (95% CI, 17.7-22.1) among those without HCV (Table 2). The unadjusted risk difference and risk ratio were 14.1% (95% CI, 8.0-20.1) and 1.71 (95% CI, 1.38-2.10), respectively. After adjusting for baseline confounding with IP weighting, the risk among those with HCV was 32.8% (95% CI, 25.6-40.7) and 21.8% (95% CI, 19.1-24.4) among those without HCV. The baseline adjusted risk difference was 11.0% (95% CI, 3.0-19.1) and the risk ratio was 1.51 (95% CI, 1.15-1.93).

**Table 2:** Estimated risks, risk differences, and risk ratios of HCV on MDR/RR-TB end-of-treatment outcomes.

**Analysis**	**Risk, % (95% CI)**		**Risk difference, % (95% CI)**	**Risk ratio (95% CI)**
	**HCV positive**	**HCV negative**		
Effect of HCV on death, failure, and loss-to-follow-up (total effect)				
Unadjusted	34.0 (28.3-39.5)	19.9 (17.7-22.1)	14.1 (8.0-20.1)	1.71 (1.38-2.10)
IP of HCV weighted	32.8 (25.6-40.7)	21.8 (19.1-24.4)	11.0 (3.0-19.1)	1.51 (1.15-1.93)
Effect of HCV on death and failure had no one been lost to follow-up (controlled direct effect)				
IP of HCV and censoring weighted	22.7 (16.0-30.6)	15.0 (12.4-17.6)	7.7 (0.8-16.2)	1.52 (1.02-2.11)
Effect of HCV on death and failure had all adhered to a strong regimen (controlled direct effect)				
IP of HCV and censoring weighted	19.9 (11.7-28.7)	12.9 (9.6-16.6)	7.0 (−1.6 to 17.3)	1.55 (0.88-2.38)

Abbreviations: HCV, hepatitis C virus; IP, inverse probability.

### Controlled direct effects of HCV on death and failure

In a pseudopopulation where no one was lost to follow-up, the risk of death and failure among those with HCV was 22.7% (95% CI, 16.0-30.6) and 15.0% (95% CI, 12.4-17.6) among those without HCV. The risk difference was 7.7% (95% CI, 0.8%-16.2%). In a pseudopopulation that adhered to a strong regimen, the risk of death and failure among those with HCV was 19.9% (95% CI, 11.7-28.7) and 12.9% (95% CI, 9.6-16.6) among those without HCV. The risk difference was 7.0% (95% CI, −1.6% to 17.3%) (Table 2). Estimates obtained using stabilized weights for the analyses described above are presented in Table S6.

## Discussion

In this study, we sought to better understand the relationship between HCV infection and outcomes of death and failure, with emphasis on two potential drivers of these outcomes: differential provider prescribing practices and medication adherence. After applying rigorous causal inference methodology to evaluate hypothetical interventions where we ensured (1) everyone adhered to a strong MDR/RR-TB regimen and (2) no one was lost-to-follow-up, we found evidence that prescribing strong regimens and supporting adequate adherence to all participants improved outcomes overall. Moreover, we found that intervening to prevent losses-to-follow-up reduced the disproportionate absolute risk experienced by those with HCV. This is evidenced by the attenuated risk difference for the controlled direct effect had no one been lost-to-follow-up compared to the total effect of HCV on unfavorable outcomes. Intervening on regimen composition and daily adherence, however, did not further mitigate the relative impact of HCV on TB treatment outcomes. Although the hypothetical intervention on losses-to-follow-up reduced the risk difference between individuals with and without HCV infection, HCV infection still had a harmful impact on MDR/RR-TB end-of-treatment outcomes.

In light of these results, additional mechanisms likely contribute to the increased risk of unfavorable outcomes in patients with HCV, like the direct adverse effects of chronic HCV disease. Globally, 70% to 80% of individuals with positive HCV serology develop active disease, and 10% to 20% of those with chronic HCV develop liver-related complications in their lifetime, including cirrhosis, hepatocellular carcinoma, and end-stage liver disease.^4^ Patients coinfected with HCV and MDR/RR-TB experience a higher incidence of hepatotoxicity than patients with HCV or MDR/RR-TB alone, supporting the hypothesis that complications of HCV disease while undergoing MDR/RR-TB treatment could be to blame for disproportionate rates of unfavorable outcomes.^5^^,^^28^ Consistent with this hypothesis, participants with HCV in our cohort had a crude greater than two-fold risk of hepatotoxicity. Notably, however, participating countries in the endTB observational study demonstrated timely detection and effective management of adverse events, which may have mitigated the impact of hepatotoxicity on treatment outcomes. In programmatic contexts with less robust monitoring, hepatotoxicity may have a greater impact on treatment outcomes. Therefore, future work should examine the extent to which hepatotoxicity mediates the relationship by comparing the risk of adverse events among people with and without HCV and examining outcome definitions that distinguish between TB-related versus non-TB related causes of death in programmatic settings.

Baseline factors associated with HCV disease, like substance use, advanced age, and male sex,^29^^-^^31^ partially explained the positive association of HCV and unfavorable MDR/RR-TB end-of-treatment outcomes. Adjustment for differences in baseline socioeconomic, demographic, and TB disease severity factors decreased the relative risks of unfavorable outcomes comparing participants with and without HCV infection (as compared to the unadjusted estimates). This indicates that a portion of the association was due to these baseline differences and highlights the importance of measurement and statistical control of these factors to reduce bias.

Given the high risk of liver-related events among coinfected patients, future research should examine whether concurrent treatment with direct-acting antivirals (DAAs) impacts outcomes. DAAs are effective at clearing HCV infection with over 95% efficacy.^32^ Several studies have found that concomitant treatment of MDR/RR-TB and HCV yielded no major safety issues,^33^ and that DAA treatment success was consistent with clinical trials and observational studies in non-TB patients.^34^^,^^35^ The WHO published a rapid communication in March 2024 suggesting, with very low certainty of evidence, that co-administration of DAAs may lead to improved TB treatment success and a slight decrease in mortality.^36^ Our study was not powered to assess whether co-administration of DAAs improves outcomes because very few participants in the endTB observational cohort received DAAs during follow-up.

Our analysis has several limitations. Classifying suboptimal adherence as <80% monthly may be too low and insufficiently granular to be meaningful in clinical practice,^37^ even though >80% adherence is the convention in TB treatment per-protocol analyses. Additionally, the determination of HCV baseline exposure mainly relied on antibody testing. HCV antibody tests, while highly sensitive for active disease, lack specificity^38^; therefore, some patients classified as antibody positive may have already cleared the infection. Despite this limitation, we observed a strong effect of HCV infection (cleared or active) on end-of-treatment outcomes. If active, but not cleared, HCV impacts TB treatment outcomes, our estimates are likely underestimates. Another limitation is the limited post-treatment follow-up data and low incidence of recurrent TB in this cohort,^39^ which constrained our ability to examine HCV’s relationship with post-treatment outcomes. We also chose to align our definition of unfavorable outcome with the WHO definition, which classify all deaths occurring during TB treatment as unfavorable outcomes, regardless of cause. Lack of consistent data on cause of death limited our ability to assess the relationship between HCV with non-TB related deaths. Moreover, the majority of exclusions were for data from Democratic People's Republic of Korea, countries with no recorded baseline HCV positive antibody test results, or unreliable adherence reporting. Our results are potentially not generalizable to countries with low prevalence of HCV due to these restrictions. Lastly, there may be residual confounding if indicators of social vulnerability did not fully capture confounding by social factors.

A key strength of this analysis is the causal inference- and intervention-based approach. By estimating controlled direct effects under hypothetical interventions, this approach enhances clinical relevance, and by considering on-treatment (time-varying) covariates in the calculation of weights, it relaxes an assumption and may improve validity. Specifically, failure to consider time-varying factors in the construction of IP of censoring weights would imply that there are no common on-treatment causes of censoring (ie, due to suboptimal adherence or a weak regimen) and end-of-treatment outcomes. However, this assumption is untenable; studies have shown that on-treatment determinants of end-of-treatment outcomes, like clinical evolution, may also influence adherence (e.g., patients feeling better or worse may decide to skip doses or prematurely default).^40^

In conclusion, we found that adjustment for baseline confounders (socioeconomic, demographic, and TB severity) reduced the relative risk of unfavorable outcomes comparing those with and without HCV, suggesting these factors partly explain the disparity. Additionally, by weighting our analytical cohort to evaluate hypothetical interventions on TB treatment factors, we found evidence that potential interventions to prevent losses-to-follow-up might further mitigate the difference in risk of death and failure comparing those with and without HCV. Future analyses should examine if interventions on HCV disease, like co-prescribing direct-acting antivirals, improve MDR/RR-TB treatment outcomes among patients with HCV.

## Ethics approval

The endTB Observational Study protocol received ethical approval from local review boards in all participating countries, as well as from the central ethics committees of the three collaborating organizations: the Partners Human Research Committee, the Médecins Sans Frontières Ethics Review Board, and the Interactive Research and Development Institutional Review Board. All participants provided written informed consent for inclusion in the observational cohort.

## Supplementary Material

Web_Material_kwag024

## Data Availability

Data will be made available on request. Requests can be submitted through the eDSI: https://endtb.org/access-endtb-data-through-edsi.
