# Supplementary material for: Estimating the effect of hepatitis C infection on multidrug-resistant tuberculosis treatment outcomes under hypothetical interventions on regimen composition and adherence
Source: Am J Epidemiol. 2026 Feb 2;195(6):1615–23. doi: 10.1093/aje/kwag024 (PMC13231843; doi:10.1093/aje/kwag024)
Supplement: Web_Material_kwag024 [file web_material_kwag024.zip › SUPPLEMENTARY_MATERIAL_AJE_revised_CLEAN_FINAL_lg new affiliation.docx]

**SUPPLEMENTARY MATERIAL**

**Estimating the effect of hepatitis C infection on multidrug-resistant tuberculosis treatment outcomes under hypothetical interventions on regimen composition and adherence**

Allison LaHood^1,2^, James Robins^3^, Helen R. Stagg^4^, Sara Sauer^2^, Saman Ahmed^5^, Mathieu Bastard^6^, Lorenzo Guglielmetti^7^, Catherine Hewison^8^, Helena Huerga^6^, Afshan K. Isani^9^, Palwasha Khan^10,11^, Uzma Khan^12^, Nino Lomtadze^13^, Shahid Mamsa^14^, Nara Melikyan^6^, Carole D. Mitnick^2,15,16^, Michael L. Rich^2,15,16^, Kwonjun Seung^2,15,16^, Muhammad Rafi Siddiqui^17^, Alena Skrahina^18^, Assel Stambekova^19^, Girum B. Tefera^20^, Molly F. Franke^2,1^

1. Department of Epidemiology, Harvard T.H. Chan School of Public Health, Boston, Massachusetts, USA
2. Department of Global Health and Social Medicine, Harvard Medical School, Boston, Massachusetts, USA
3. CAUSALab, Department of Epidemiology, Harvard T.H. Chan School of Public Health, Boston, Massachusetts, USA
4. Department of Infectious Disease Epidemiology, London School of Hygiene & Tropical Medicine, London, United Kingdom
5. Interactive Research and Development (IRD), Karachi, Pakistan
6. Field Epidemiology Department, Epicentre, Paris, France
7. Department of Infectious, Tropical Diseases and Microbiology, IRCCS Sacro Cuore Don Calabria Hospital, Negrar di Valpolicella, Verona, Italy
8. Médecins Sans Frontières, Paris, France
9. Centers for Disease Control and Prevention, Directorate General Health Services, Sindh, Pakistan
10. Interactive Research and Development (IRD) Global, Singapore
11. Department of Clinical Research, Faculty of Infectious and Tropical Diseases, London School of Hygiene & Tropical Medicine, London, United Kingdom
12. Department of Epidemiology, Biostatistics, and Occupational Health, McGill University, Montreal, Canada
13. The National Center for Tuberculosis and Lung Diseases, Tbilisi, Georgia
14. Indus Hospital & Health Network (IHHN), Karachi, Pakistan
15. Partners In Health, Boston, Massachusetts, USA
16. Division of Global Health Equity, Brigham and Women's Hospital, Boston, Massachusetts, USA
17. Institute of Chest Diseases (ICD), Kotri, Sindh, Pakistan
18. Republican Scientific and Practical Center of Pulmonology and Tuberculosis, Minsk, Belarus
19. Partners In Health, Kazakhstan
20. Partners In Health, Freetown, Sierra Leone

**Table of Contents**

Figure S1 Page 2

Tables S1-S2 Page 2

Tables S3-S4 Page 3

Table S5 Page 4

Table S6 Page 5


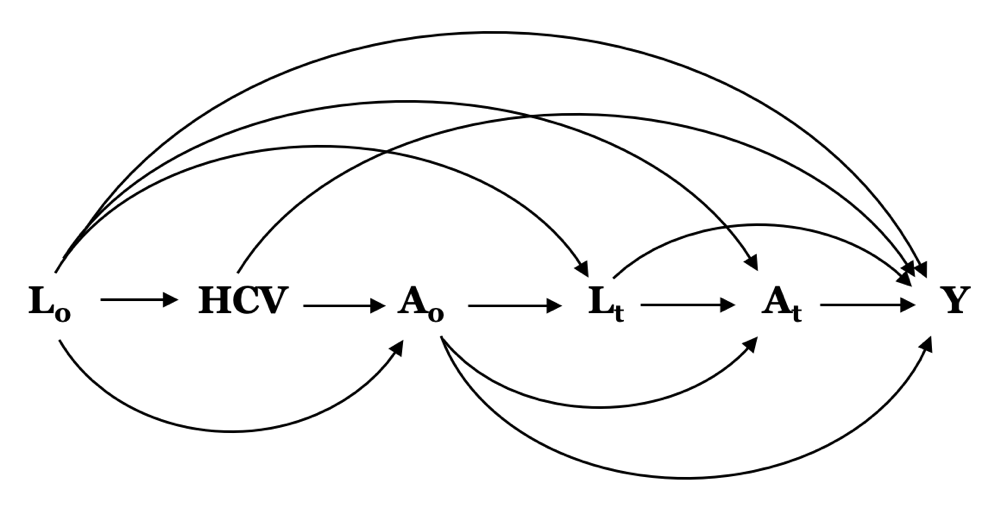


***Figure S1.*** *Directed acyclic graph depicting relationships between analysis covariates.* ***HCV****: hepatitis C infection.* ***L_0_:*** *sex, age, HIV, low BMI, intravenous drug use, alcohol use, unemployment, partnership status, baseline cavitary disease, baseline sputum smear, baseline culture.* ***A_t_:*** *Loss-to-follow-up or adequate adherence to strong MDR/RR-TB regimen, depending on the controlled direct effect of interest.* ***L_t_:*** *sputum smear, culture, treatment month, quadratic treatment month, cavitary disease.* ***Y:*** *MDR/RR-TB end-of-treatment outcome*

***Table S1.*** *Distribution of included participants by study country (N = 1,530).*

| **Characteristic** | **Overall**,  N = 1,530 |
| --- | --- |
|  |  |
|  | n (%) |
| Armenia | 95 (6.2%) |
| Belarus | 102 (6.7%) |
| Ethiopia | 41 (2.7%) |
| Georgia | 238 (16%) |
| Indonesia | 58 (3.8%) |
| Kazakhstan | 656 (43%) |
| Myanmar | 43 (2.8%) |
| Pakistan | 265 (17%) |
| Vietnam | 32 (2.1%) |

***Table S2.*** *MDR/RR-TB end-of-treatment outcomes among eligible participants (N = 1,530).*

| **End-of-treatment outcome** | **HCV test results** | | **Overall,**  **n (%)**  N = 1,530 |
| --- | --- | --- | --- |
|  | **Positive,**  **n (%)**  N = 256 | **Negative,**  **n (%)**  N = 1,274 |  |
| Completed | 14 (5.5%) | 65 (5.1%) | 79 (5.2%) |
| Cured | 155 (61.0%) | 956 (75.0%) | 1,111 (72.6%) |
| Died | 31 (12.0%) | 92 (7.2%) | 123 (8.0%) |
| Failed | 22 (8.6%) | 65 (5.1%) | 87 (5.7%) |
| Lost-to-follow-up | 34 (13.0%) | 96 (7.5%) | 130 (8.5%) |

**Abbreviations:** Hepatitis C virus (HCV)

***Table S3.*** *Clinician-reported reasons for treatment failure among participants who failed treatment in the analytic cohort (N = 87).*

| **Reason for treatment failure** | **HCV test results** | | **Overall,**  **n (%)**  N = 87 |
| --- | --- | --- | --- |
|  | **Positive,**  **n (%)**  N = 22 | **Negative,**  **n (%)**  N = 65 |  |
| Adverse drug reactions | 4 (18.2%) | 6 (9.2%) | 10 (11.5%) |
| Microbiologic failure | 15 (68.2%) | 52 (80.0%) | 67 (77.0%) |
| No radiological improvement | 0 (0.0%) | 3 (4.6%) | 3 (3.4%) |
| Other | 2 (9.1%) | 0 (0.0%) | 2 (2.3%) |
| Unknown | 1 (4.5%) | 4 (6.2%) | 5 (5.7%) |

**Abbreviations:** Hepatitis C virus (HCV)

***Table S4.*** *Univariate association between HCV infection and hepatotoxicity.*

| **Exposure** | **Outcome** | **Risk Ratio (95% CI)** |
| --- | --- | --- |
| HCV infection | Hepatotoxicity* | 2.24 (1.43, 3.20) |

**Abbreviations:** Hepatitis C virus (HCV)

*Hepatotoxicity defined as baseline liver injury or on-treatment hepatotoxicity (grade ≥3)

***Table S5.*** *Specification of inverse probability of treatment weights and time-varying inverse probability of censoring weights.*

| **Specification** | **Weight** | **Mean (SD)** | **Min, max** |
| --- | --- | --- | --- |
| ***Inverse probability of HCV weights to adjust for baseline confounding*** | | | |
| Numerator includes HCV (stabilized). Denominator includes baseline confounders*. | Unstabilized | 2.02 (3.27) | 1.01, 40.90 |
|  | Stabilized | 1.00 (0.54) | 0.18, 7.05 |
| ***Inverse probability of censoring weights for censoring due to loss-to-follow-up*** | | | |
| Numerator includes treatment month, quadratic treathment month, HCV, and baseline confounders* (stabilized). Denominator includes time, quadratic time, HCV, baseline confounders*, time-varying smear result (+/-), time-varying culture result (+/-), and time-varying cavitary disease (0/1). | Unstabilized | 1.09 (0.08) | 1.00, 1.84 |
|  | Stabilized | 1.00 (0.04) | 0.74, 1.55 |
| ***Inverse probability of censoring weights for censoring due to inadequate adherence to strong MDR/RR-TB regimens*** | | | |
| Numerator includes treatment month, quadratic treatment month, HCV, and baseline confounders* (stabilized). Denominator includes time, quadratic time, HCV, baseline confounders*, time-varying smear result (+/-), time-varying culture result (+/-), and time-varying cavitary disease (0/1). | Unstabilized | 2.02 (0.82) | 1.21, 14.0 |
|  | Stabilized | 1.00 (0.18) | 0.55, 3.68 |

**Abbreviations**: Hepatitis C virus (HCV), standard deviation (SD), rifampin-resistant/multidrug-resistant tuberculosis (MDR/RR-TB)

*baseline confounders include sex, age, HIV, low BMI (<18.5), intravenous drug use, alcohol use, unemployment, partnership, smear result (+/-), culture result (+/-), cavitary disease (0/1), and study country with considerable missingness in adherence data

***Table S6.*** *Estimated risks, risk differences, and risk ratios of HCV on MDR/RR-TB end-of-treatment outcomes in primary analyses including stabilized and unstabilized weighted estimates.*

| **Analysis** | **Risk, % (95% CI)** | | **Risk Difference,**  **% (95% CI)** | **Risk Ratio**  **(95% CI)** |
| --- | --- | --- | --- | --- |
|  | **HCV positive** | **HCV negative** |  |  |
| ***Effect of HCV on death, failure, and loss-to-follow-up (total effect)*** | | | | |
| Unadjusted | 34.0 (28.3, 39.5) | 19.9 (17.7, 22.1) | 14.1 (8.0, 20.1) | 1.71 (1.38, 2.10) |
| IP of HCV weighted,  unstabilized | 32.8 (25.6, 40.7) | 21.8 (19.1, 24.4) | 11.0 (3.0, 19.1) | 1.51 (1.15, 1.93) |
| IP of HCV weighted,  stabilized | 32.4 (25.2, 40.4) | 21.2 (18.8, 23.7) | 11.2 (3.2, 19.2) | 1.53 (1.16, 1.95) |
| ***Effect of HCV on death and failure had no one been lost-to-follow-up (controlled direct effect)*** | | | | |
| IP of HCV and censoring weighted,  unstabilized | 22.7 (16.0, 30.6) | 15.0 (12.4, 17.6) | 7.7 (0.8, 16.2) | 1.52 (1.02, 2.11) |
| IP of HCV and censoring weighted,  stabilized | 22.7 (16.0, 30.2) | 15.4 (13.0, 17.7) | 7.3 (0.6, 15.4) | 1.53 (1.03, 2.29) |
| ***Effect of HCV on death and failure had all adhered to a strong regimen (controlled direct effect)*** | | | | |
| IP of HCV and censoring weighted,  unstabilized | 19.9 (11.7, 28.7) | 12.9 (9.6, 16.6) | 7.0 (-1.6, 17.3) | 1.55 (0.88, 2.38) |
| IP of HCV and censoring weighted,  stabilized | 21.0 (12.3, 31.1) | 14.2 (10.6, 17.1) | 6.8 (-3.1, 16.6) | 1.62 (0.83, 3.38) |

**Abbreviations:** Hepatitis C virus (HCV), Inverse probability (IP)
